# A Sequential Ultrafiltration Method to Enhance the Accuracy and Throughput in Plasma Protein Binding Tests

**DOI:** 10.3390/pharmaceutics17020273

**Published:** 2025-02-18

**Authors:** Sang Ho Jeon, Min Chang Kim, Haejun Lee, Ju-Hee Oh, Hyun Seo Kim, Heawon Lee, Taehoon Park, Young-Joo Lee

**Affiliations:** 1 Department of Biomedical and Pharmaceutical Sciences, Kyung Hee University, 1 Hoegi-dong, Dongdaemun-gu, Seoul 130-701, Republic of Korea; sangho9648@khu.ac.kr (S.H.J.); mcmr1218@khu.ac.kr (M.C.K.); cj_xellos2@khu.ac.kr (H.L.); go7469@khu.ac.kr (H.L.); neofriend@khu.ac.kr (T.P.); 2 Division of Biopharmaceutics, College of Pharmacy, Kyung Hee University, 1 Hoegi-dong, Dongdaemun-gu, Seoul 130-701, Republic of Korea; 5juhee@khu.ac.kr (J.-H.O.); kimsky3@khu.ac.kr (H.S.K.); 3 Department of Integrated Drug Development and Natural Products, Kyung Hee University, 1 Hoegi-dong, Dongdaemun-gu, Seoul 130-701, Republic of Korea

**Keywords:** plasma protein binding, ultrafiltration, non-specific binding

## Abstract

**Objectives**: Ultrafiltration (UF) is widely accepted as a method for assessing the plasma protein binding (PPB) of drugs. However, it is vulnerable to non-specific binding (NSB) to the device, which can result in inaccuracies. This study presents a straightforward, high-throughput modified UF method aimed at minimizing bias due to NSB. **Methods**: The modified UF method, sequential UF, features the addition of a 2 min pre-UF phase designed to saturate the NSB in the device, followed by the main 20 min UF procedure, compared to the conventional UF method. To evaluate the feasibility of this sequential UF method, we measured the PPB of nine compounds using sequential UF and compared these results to those obtained with the conventional mass balance UF method, recognized as a standard for NSB correction. **Results**: The PPB values determined through sequential UF were generally consistent with those derived from the mass balance UF method. The fold differences ranged from 97.9% to 113.8%, with an average of 103.5%. No significant differences were observed between the two methods for all compounds, with the exception of quercetin, which showed an unusually high PPB. **Conclusions**: Sequential UF was effective in correcting NSB to the device while providing advantages in terms of simplicity and efficiency.

## 1. Introduction

**Figure 1:**
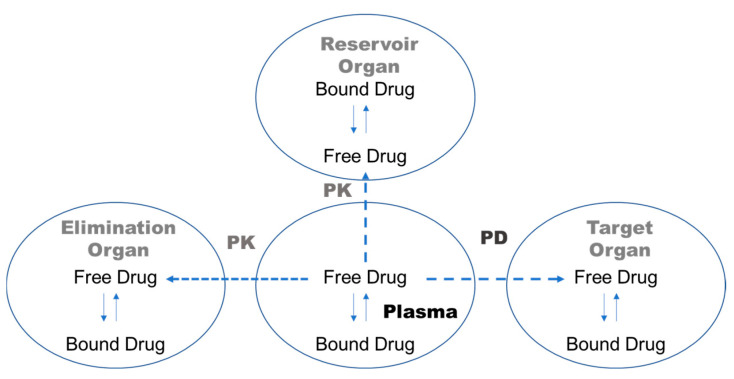
Schematic diagram illustrating how the free drug fraction in plasma influences drug pharmacokinetics and pharmacodynamics.

When drugs are administered, they bind to plasma proteins, such as albumin, α1-acid glycoprotein, or lipoproteins, with different affinity and kinetics [1,2]. Only unbound, free drugs can pass through biological membranes [1], so the free drug concentration in plasma is related to the free drug concentration at the receptor site, which determines the pharmacological activity of a drug (Figure 1) [2]. From a pharmacokinetic perspective, the degree of binding to both plasma proteins and tissue components affects the volume of distribution in a steady state. In addition, drug clearance can be considerably affected by the degree of protein binding of the drug [3]. Thus, highly protein-bound drugs could have lower systemic clearance and longer half-lives, with limited distribution to tissues when other physicochemical conditions are similar, compared with low-protein-bound drugs [3]. Consequently, plasma protein binding (PPB) emerges as a pivotal parameter in the pharmacokinetics of drug distribution, metabolism, and excretion (Figure 1) [4,5].

The commonly employed methods for measuring PPB include ultracentrifugation, equilibrium dialysis, and ultrafiltration (UF) [6,7,8]. Ultracentrifugation is based on the premise of varying sedimentation velocities that correlate with the weight of molecules in a solvent. This technique necessitates prolonged high-speed spinning, often approximately six hours. During this interval, protein-bound drugs sediment at the bottom of the tube, whereas unbound drugs remain in the supernatant. Conversely, equilibrium dialysis utilizes a device comprising two compartments separated by a semipermeable membrane. Following an incubation period of roughly three to forty-eight hours, the first compartment contains only the protein-bound drug, while the unbound drug is isolated within the buffer solution of the second compartment. While both methods provide valuable insights into protein interactions and ensure robust experimental conditions for equilibrium dialysis and negligible non-specific binding (NSB) to membranes for ultracentrifugation, they require considerable time investment [1].

UF employs a filtering membrane to differentiate between protein-bound and unbound drugs through the application of positive pressure or centrifugation [9]. This method is prevalent for measuring drug PPB due to its high-throughput capabilities and relatively straightforward experimental design [7]; however, it can be influenced by non-specific binding (NSB) to the filter membrane [8].

To mitigate NSB in the UF procedure, several modified techniques have been proposed. The prewashing of the UF membranes with surfactants such as 5% benzalkonium chloride or 0.5% to 5% Tween-80, in addition to utilizing control plasma samples in parallel [10,11], the modification of UF membranes to enhance selectivity and reduce fouling [12], and the optimization of operational conditions [7,13] have been suggested. The mass balance UF method is often preferred for addressing methodological challenges associated with NSB in the UF procedure, utilizing a mathematical balance between the retentate and filtrate to minimize the effects of NSB [14]. Nevertheless, this approach can also be time-consuming, necessitating sample weighing, analysis across a wide concentration range, and the assumption of equal volume density between the retentate and filtrate, which may not be realistically achievable.

This study developed a modified UF method (sequential UF) aimed at reducing the impacts of NSB by simple changes in the experimental procedures. The developed method was employed to investigate the PPB of nine pharmacological compounds, and the results were compared to those derived from mass balance UF to evaluate the feasibility of this method.

## 2. Materials and Methods

### 2.1. Materials

Centrifugal filter units (Amicon^®^ Ultra 0.5 mL with Ultracel^®^; 30 K molecular weight cutoff) were purchased from Millipore (Burlington, MA, USA), and ciclopirox, irinotecan hydrochloride, quercetin, and rhodamine 123 were purchased from Sigma-Aldrich (St. Louis, MO, USA). SN-38 was supplied by TCI (Tokyo, Japan). Atorvastatin and otaplimastat were kindly donated by CJ Healthcare (Seoul, Republic of Korea). New drug candidates for cancer, identified as DW1901 and DW1902, were provided by Daewoo Pharm Co., Ltd. (Busan, Republic of Korea). Otaplimastat, a new drug candidate for stroke, was supplied by Shipoong Co., Ltd. (Seoul, Republic of Korea). All other chemicals and solvents were obtained commercially. Rat plasma was collected from male Sprague Dawley rats purchased from Orient Bio Inc. (Seongnam, Republic of Korea).

### 2.2. Plasma Sample Preparation

Rat plasma was collected from male Sprague Dawley rats purchased from Orient Bio Inc. (Seongnam, Republic of Korea). The plasma was separated from whole blood samples using centrifugation and was utilized immediately for the PPB test.

For the PPB study, plasma samples containing rhodamine 123, quercetin, otaplimastat, atorvastatin, ciclopirox, irinotecan, SN-38, DW1901, and DW1902 were prepared. All compounds, with the exception of otaplimastat and ciclopirox, were added to 1 mL of blank plasma to achieve a drug concentration of 10 μM. Otaplimastat and ciclopirox were examined at concentrations of 5 μM and 1 μg/mL, respectively. The compound-added plasma samples were incubated in a water bath at 37 °C and agitated at 50 rpm for two hours prior to the PPB test.

### 2.3. Conventional UF with and Without NSB Correction

The device used in the experiment was Amicon^®^ Ultra, which is commonly used in UF experiments [15]. UF was conducted following the manufacturer’s user guide. Briefly, the centrifugal filter units were washed once with 0.1N NaOH and then twice with double-distilled water by centrifugation at 14,000× *g* for 15 min. After loading 0.5 mL of the plasma sample onto the filter of the filtrate collection tube (FT tube), the tube was centrifuged at 14,000× *g* for 2 min at 25 °C [16]. The exact process was performed using phosphate-buffered saline (PBS) instead of plasma to obtain a filtrate solution for correction with PBS [13].

The percentage PPB was calculated using Equation (1) for conventional UF without NSB correction:

(1) $$\%\;PPB=\left\{\frac{C_{S}-C_{F}}{C_{S}}\right\}\times 100,$$

where C_s_ is the concentration of the plasma sample, and C_F_ represents the concentration of the filtrate sample obtained from the ultrafiltration process.

In instances where NSB correction was applied, the percentage PPB was computed using Equation (2) [14]:

(2) $$\%\;PPB=\left\{\frac{C_{FB}/C_{SB}-C_{F}/C_{S}}{C_{FB}/C_{SB}}\right\}\times 100$$

In this equation, C_SB_ indicates the concentration of the buffer sample, while C_FB_ signifies the concentration of the filtrate sample derived from ultrafiltration using the buffer.

### 2.4. Mass Balance UF

**Figure 2:**
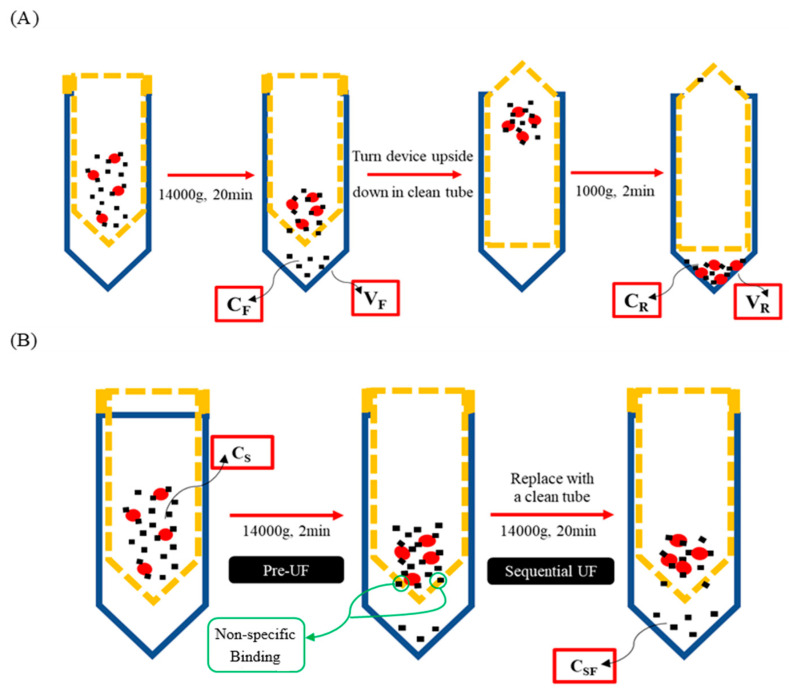
Illustration of mass balance UF (**A**) and sequential UF (**B**). Blue, FT or RT tube; yellow dotted line, filter device; red circle, plasma protein; small black square, testing compound.

Washing procedures were performed in accordance with established methods for conventional ultrafiltration. Further procedures were conducted as per the protocols outlined by Wang and Williams [14]. In brief, 0.5 mL of the plasma sample was introduced onto the filter of the filtrate tube (FT tube) and subsequently subjected to centrifugation at 14,000× *g* for 20 min at 25 °C. Following centrifugation, the filter was carefully detached from the FT tube, and the filtrate was removed. The filter was then inverted and transferred to a new retentate collection tube (RT tube), where it underwent a second centrifugation at 1000× *g* for 2 min at 25 °C. The retentate was subsequently extracted from the RT tube (Figure 2A).

The percentage PPB was calculated using Equation (3) for mass balance UF:

(3) $$\%\;PPB=\left\{\frac{\left(C_{R}-C_{F}\right)\times V_{R}}{\left(C_{R}\times V_{R}+C_{F}\times V_{F}\right)}\right\}\times 100$$

In this equation, C_R_ and V_R_ represent the concentration and volume of the retentate obtained from the final RT tube, while C_F_ and V_F_ denote the concentration and volume of the filtrate collected from the FT tube. The values for V_R_ and V_F_ were determined based on the weights of the retentate and filtrate, with the assumption that both exhibit a density of 1 g/mL [14].

### 2.5. Sequential UF

A volume of 0.5 mL of the plasma sample was initially loaded onto the filter within the FT tube. The tube was then subjected to centrifugation at 14,000× *g* for 2 min at a temperature of 25 °C, serving as the pre-ultrafiltration (pre-UF) step. Following this pre-UF process, the filter was transferred to a new FT tube and centrifuged a second time at 14,000× *g* for 20 min at 25 °C to collect the filtrate sample (Figure 2B).

The percentage PPB was calculated using Equation (4) for sequential UF:

(4) $$\%\;PPB=\left\{\frac{C_{S}-C_{SF}}{S}\right\}\times 100,$$

where C_s_ represents the concentration of the plasma sample, and C_SF_ denotes the concentration of the filtrate sample obtained from the sequential ultrafiltration conducted for 20 min.

### 2.6. Determination of Optimal Pre-UF Time for Sequential UF

Initially, 0.5 mL of the plasma sample was loaded onto the filter of the FT tube to obtain a baseline plasma sample at 0 min. Subsequently, the tube was centrifuged at 14,000× *g* for 0.5, 1, 2, and 5 min at 25 °C, resulting in the collection of both plasma and filtrate samples. For this investigation, otaplimastat and rhodamine 123 were utilized as the study compounds.

### 2.7. Quantitative Analysis

The concentration of rhodamine 123 in samples was quantified following a modified protocol based on the work of Vora [17]. Briefly, a high-performance liquid chromatography system equipped with a Phenomenex Luna column (25 cm × 4.6 mm, i.d. particle size 5 µm; Torrance, CA, USA) was used. The mobile phase was a mixture of 50 mM phosphate buffer and acetonitrile (65:35, *v*/*v*) adjusted to pH 3.2 with phosphoric acid. Detection was performed using a fluorescence detector (480 nm for excitation and 530 nm for emission). For the analysis of other compounds, a Waters Quattro micro™ API mass spectrometer (Waters Corp., Milford, MA, USA) equipped with an ACUITY ultraperformance liquid chromatography (UPLC^®^) system was used. The specific conditions for UPLC and mass spectrometry for each analyzed compound are detailed in Table 1. The validation procedure followed the latest FDA guidelines.

### 2.8. Comparison of Methods

The PPB of atorvastatin, ciclopirox, DW1901, DW1902, irinotecan, otaplimastat, rhodamine 123, SN-38, and quercetin was assessed utilizing both the mass balance UF and sequential UF methodologies. The compounds used in the evaluation were randomly selected from those for which analytical method validation had been completed in the laboratory, ensuring a diverse range of lipid solubility. The bound and unbound plasma fractions were subjected to linear regression analysis, with the regression line established by constraining the y-intercept to zero [14]. The fold difference in PPB between the different methods was computed using Equation (5):

(5) $$Fold\;difference=\frac{\%\;PPB\;by\;sequence\;UF}{\%\;PPB\;by\;mass\;balance\;UF}$$

### 2.9. Statistical Analysis

PPB data were obtained from a minimum of three independent experiments. The results are expressed as the mean ± standard deviation. An unpaired *t*-test with unequal variance was applied to compare the means of the two independent groups, while Levene’s test was conducted to evaluate the equality of variances between the two methodologies. A *p* value of less than 0.05 was deemed statistically significant.

## 3. Results

### 3.1. Optimal Pre-UF Time

**Figure 3:**
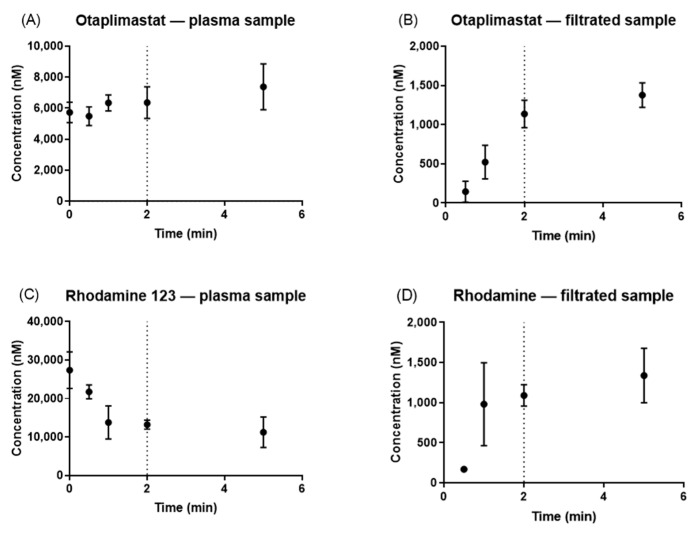
Time–concentration profile of otaplimastat, rhodamine 123, and atorvastatin. The concentration of the plasma sample and filtrated sample was plotted: otaplimastat (**A**,**B**), and rhodamine 123 (**C**,**D**).

In comparing the concentrations of the plasma samples with those of the filtrated samples, it was observed that otaplimastat and rhodamine 123 achieved a state of equilibrium between the 1 min and 2 min intervals (Figure 3). Therefore, the pre-UF time was established at 2 min, given that NSB had already reached saturation by this time point.

### 3.2. Effects of NSB on the PPB of Rhodamine 123

**Figure 4:**
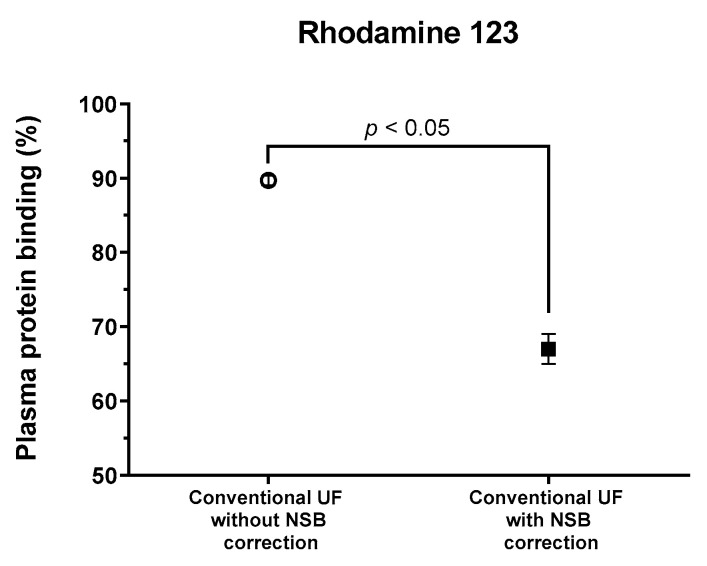
The PPB of rhodamine 123 by conventional UF was affected by the presence or absence of NSB correction (unpaired *t*-test, *p* < 0.05).

The percentage of PPB of rhodamine 123 measured through conventional UF without NSB correction was determined to be 89.68% ± 0.63%. Conversely, the PPB measured using UF with NSB correction facilitated by phosphate-buffered saline (PBS) was significantly lower, at 67.02% ± 2.03% (*p* < 0.05, Figure 4). The protein binding obtained after NSB correction was comparable to the literature value of 70% obtained through equilibrium dialysis [25].

### 3.3. Comparison of Mass Balance UF and Sequential UF

**Figure 5:**
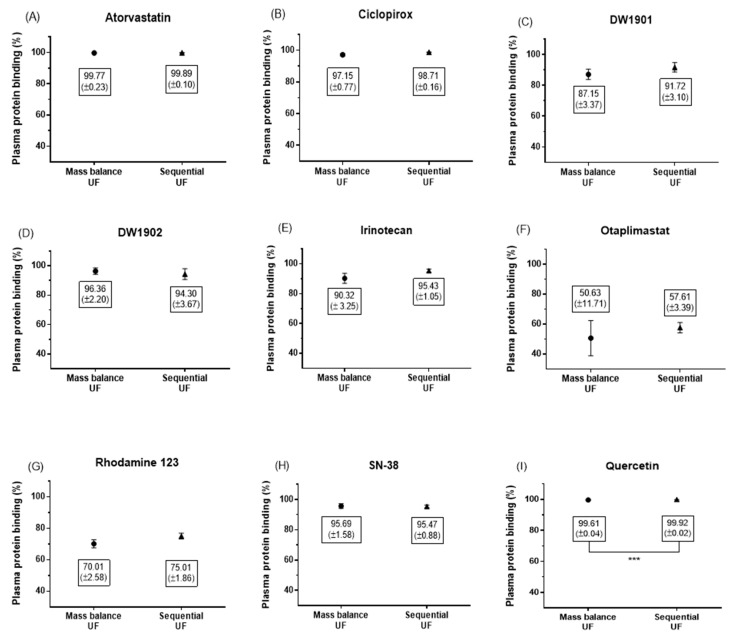
Differences between sequential UF and mass balance UF were statistically identified using an unpaired *t*-test for nine compounds: atorvastatin (**A**), ciclopirox (**B**), DW1901 (**C**), DW1902 (**D**), irinotecan (**E**), otaplimastat (**F**), Rhodamine 123 (**G**), SN-38 (**H**), and quercetin (**I**). There was no significant difference in the PPB of all compounds, except quercetin, between the two methods (unpaired *t*-test, *p* > 0.05). Data were obtained from a minimum of three independent experiments.

In this study, we compared the percentage of PPB for nine compounds using two different methods: sequential UF and mass balance UF. The results for PPB are summarized in Figure 5.

The fold differences in PPB across the compounds ranged from 97.9% to 113.8%, with a mean of 103.5%. Notably, there were no statistically significant differences between the two methods for all compounds, with the exception of quercetin, which showed a PPB of 99.92% ± 0.02% by sequential UF, significantly differing from 99.61% ± 0.04% by mass balance UF (unpaired *t*-test, *p* = 0.0006; see Figure 5I). However, the absolute difference was minimal at just 0.31%.

**Figure 6:**
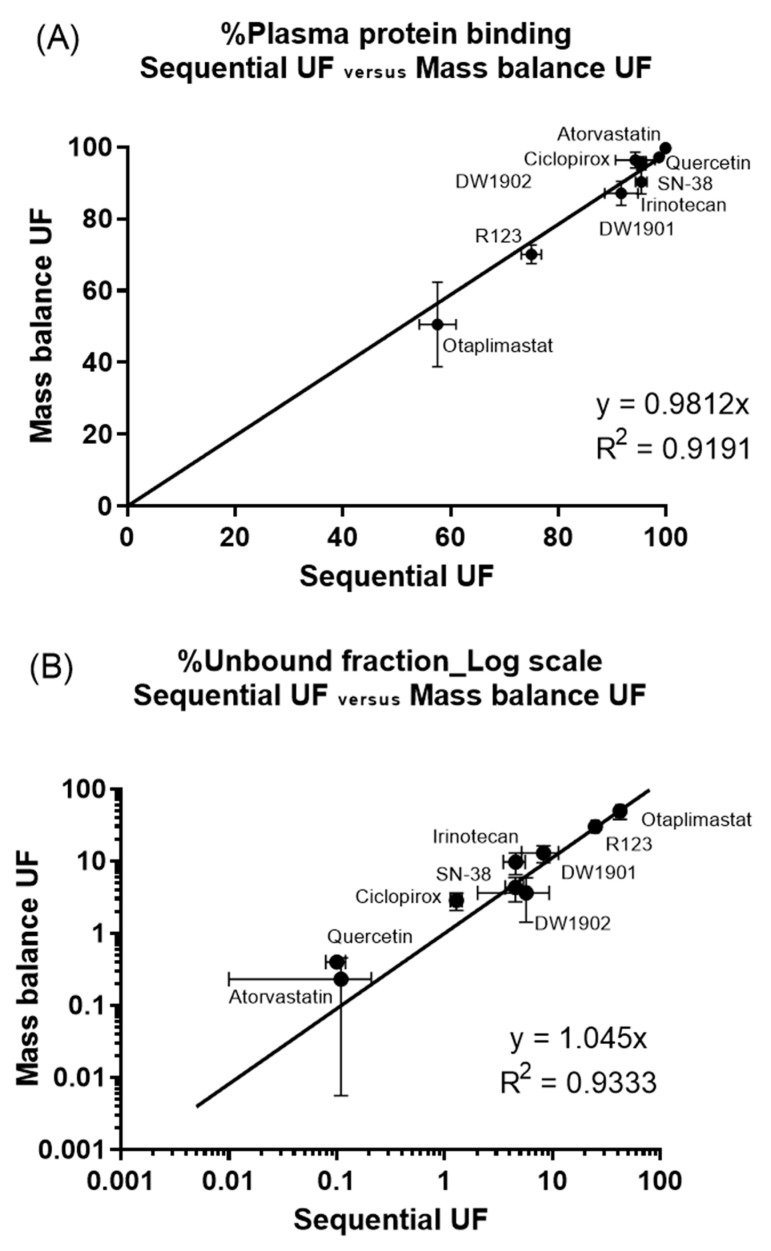
Correlation of PPB by sequential UF and mass balance UF: (**A**) bound fraction and (**B**) unbound fraction. The unbound fraction was plotted with the logarithmic scale to express high protein binding.

Furthermore, a strong correlation was observed between the bound and unbound fractions of the nine compounds for each method. The linear regression slope for the bound fraction was 0.9812 with an R^2^ value of 0.9191, while the unbound fraction had a slope of 1.045 with an R^2^ of 0.9333 (Figure 6). These findings suggest that sequential UF could serve as a viable alternative to mass balance UF for evaluating plasma protein binding.

### 3.4. Comparison of Variance in Results

**Figure 7:**
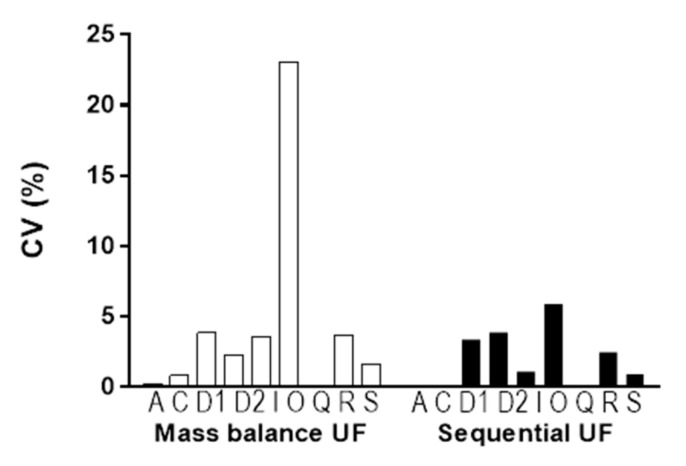
Comparison of the CV% of PPB by mass balance UF (□) and sequential UF (■). Each bar represents the CV% of the PPB of atorvastatin (A), ciclopirox (C), DW1901 (D1), DW1902 (D2), irinotecan (I), otaplimastat (O), quercetin (Q), rhodamine 123 (R), and SN-38 (S) from left to right.

The coefficient of variation (CV%) for PPB obtained through mass balance UF (□) and sequential UF (■) was calculated to compare the variance in each method (Figure 7). The CV% for sequential UF was smaller than that for mass balance UF. Levene’s test for the equality of variances of each method showed a significant difference (*p* < 0.05).

## 4. Discussion

The PPB characteristics of drugs have a profound impact on their pharmacokinetic and pharmacodynamic profiles. Consequently, evaluating the protein binding of novel chemical entities in the early stages of drug development is essential for clarifying their absorption, distribution, metabolism, and excretion properties. This assessment is instrumental in informing the lead optimization process [26,27].

UF has emerged as a widely adopted method for estimating protein binding, largely due to its methodological simplicity and the lack of necessity for expensive equipment, such as ultracentrifuges [9,13]. However, one significant limitation of UF is the potential for the adsorption of test compounds onto filter membranes, which can lead to inaccurate binding estimations. Therefore, it is imperative to develop strategies to either mitigate or calibrate this adsorption effect to enhance the reliability of protein binding data [16].

In our analysis, the PPB of rhodamine 123, when calculated via conventional UF without considering non-specific binding (NSB) correction, was found to exceed the PPB determined with NSB correction using PBS by 22.6%. While PBS correction provides a viable approach to address NSB, it is important to note that the extent of membrane adsorption can vary between plasma and PBS for specific compounds, indicating that this method has inherent limitations [14].

Mass balance UF represents a more robust alternative for addressing UF-related adsorption challenges; however, it entails a more intricate experimental design [14]. In this study, we introduced a sequential UF method designed to minimize NSB with reduced procedural complexity. The core of the sequential UF process involves an initial pretreatment phase aimed at saturating the filter membrane adsorption. By segmenting a single 20 min UF procedure into a 2 min pre-UF phase followed by a 20 min main UF phase, we postulated that the saturation of adsorption would occur during the pre-UF phase, rendering the adsorption effect negligible in the subsequent main UF process. Our findings indicated that the results from these two methodologies, the proposed sequential UF and mass balance UF, were comparable. With the exception of quercetin, no significant differences in PPB were observed between the approaches. Even in the case of quercetin, the absolute discrepancy was merely 0.31%, suggesting that the statistical significance likely arose from small variations in the standard deviation.

**Figure 8:**
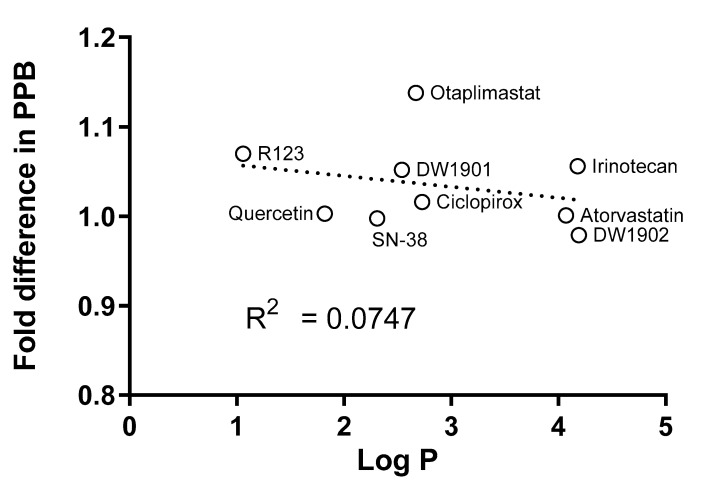
A correlation between log *p* and the fold difference in PPB was not observed (*R*^2^ = 0.0747). The fold difference between the two methods was calculated using Equation (5).

The degree of lipophilicity influences the binding of drugs to the filter membrane [7,13], and it is known that it is difficult to determine the plasma protein binding of many investigated compounds due to their high lipophilic properties [14]. Therefore, we evaluated whether lipophilicity continues to impact the outcomes of sequential UF. The correlation between the fold difference in PPB and lipophilicity, as expressed by log *p*, was weak, with an R^2^ value of 0.0747 (Table 2 and Figure 8). This non-correlation implies that the observed differences may be attributed to random experimental errors rather than systemic biases linked to lipophilic characteristics and NSB.

Encouragingly, we observed a one-to-one correspondence in the mean bound and unbound fractions derived from both methods, reinforcing the notion that sequential UF presents a straightforward and high-throughput alternative to mass balance UF (Figure 6). Furthermore, when comparing sequential UF to mass balance UF, several advantages emerge. First, sequential UF eliminates the need for weighing (Figure 2). Second, the number of input variables required for PPB calculation is halved. Mass balance UF necessitates parameters such as C_F_, C_R_, V_F_, and V_R_, while sequential UF simplifies this to merely C_S_ and C_SF_ (Figure 2). Assuming that C_S_ is constant and predetermined in the experimental setup reduces the complexity further, as only C_SF_ is needed to estimate PPB. The minimal V_R_ may also contribute to potential experimental error. Furthermore, the assumption of equal density for V_F_ and V_R_ is no longer required, which analytically cannot be justified in all cases. Collectively, these factors contribute to a lower coefficient of variation (CV%) for PPB results acquired through sequential UF compared to those obtained via mass balance UF (Figure 7).

Moreover, sequential UF alleviates the analytical workload associated with the process. Significant discrepancies between C_R_ and C_F_, particularly for compounds exhibiting high PPB, can often occur. As exemplified by quercetin, a 1000-fold difference was found between these values, complicating the validation process for the analytical method across a broad concentration range. Given that C_S_ is typically lower than C_R_ and can sometimes be employed as a fixed parameter, the analytical burden for sequential UF appears comparatively smaller.

Further discussion is needed to assess whether the sequential UF method can be applied in situations where precise figures are essential, such as submissions to regulatory authorities. This is important to note because the method has the inherent limitations of UF methods. Consequently, the sequential UF method is anticipated to be most effective in high-throughput protein binding tests during the early stages of new drug development. However, further investigation is required to determine how accurately sequential UF can be applied to various devices with different molecular weight cutoffs.

## 5. Conclusions

Despite the challenges associated with NSB, UF is widely used in new drug development. Sequential UF is designed to be a straightforward and efficient method for PPB. Unlike previous modified techniques that aimed to reduce NSB, sequential UF offers the advantage of simplicity in the PPB evaluation process and its associated analytical setup process. The benefits of sequential UF are particularly significant in applications such as high-throughput screening for drug-like properties, making it a viable alternative to mass balance ultrafiltration techniques.

## Figures and Tables

**Table 1:** LC-MS/MS conditions of the test compounds.

Compounds	UPLC Condition			MS		Reference
				Condition		
	Column	Mobile Phase		Ionization	Multiple Reaction Monitoring Transition	
				Mode	(*m*/*z*)	
Atorvastatin	C18	(A) Acetonitrile		[M + H]^+^	559.7 > 440.0	[18]
		(B) 0.1% formic acid in distilled water, 70:30 (*v*/*v*)				
Ciclopirox	C18	(A) 0.1% formic acid in 50% acetonitrile		[M + H]^+^	222.0 > 103.7	[19]
		(B) Isopropanol, 90:10 (*v*/*v*)				
DW1901	C18	(A) Acetonitrile		[M + H]^+^	331.0 > 141.9	[20]
		(B) 0.1% formic acid in distilled water, 85:15 (*v*/*v*)				
DW1902	C18	(A) Acetonitrile		[M + H]^+^	358.3 > 109.0	[20]
		(B) 0.1% formic acid in distilled water, 85:15 (*v*/*v*)				
Irinotecan	C18	(A) 0.1% formic acid in acetonitrile		[M + H]^+^	393.3 > 349.1	[21]
		(B) 0.1% formic acid in distilled water, gradient				
Otaplimastat	C18	(A) Acetonitrile		[M + H]^+^	535.2 > 203.0	[22]
		(B) 10mM ammonium acetate, 60:40 (*v*/*v*)				
SN-38	C18	(A) 0.1% formic acid in acetonitrile		[M + H]^+^	349.3 > 305.1	[23]
		(B) 0.1% formic acid in distilled water, gradient				
Quercetin	C18	(A) 0.1% acetic acid in acetonitrile		[M − H]^−^	301.0 > 151.0	[24]
		(B) 1mM ammonium acetate (pH 2.5 with acetic acid), 70:30 (*v*/*v*)				

**Table 2:** Log *p* value of the test compounds.

Compound	Log *p*	Reference
Atorvastatin	4.07	[28]
Ciclopirox	2.73	[29]
DW1901	2.54	^a^
DW1902	4.19	^a^
Irinotecan	4.18	[30]
Otaplimastat	2.67	^b^
Quercetin	1.82	[31]
Rhodamine 123	1.06	[32]
SN-38	2.31	[30]

^a^ Calculated log *p* using Marvin supported by Chemaxon. ^b^ In-house unpublished data.

## Data Availability

The original contributions presented in this study are included in the article. Further inquiries can be directed to the corresponding author.
